# Investigating the accuracy of Apple Watch VO_2_ max measurements: A validation study

**DOI:** 10.1371/journal.pone.0323741

**Published:** 2025-05-15

**Authors:** Rory Lambe, Ben O’Grady, Maximus Baldwin, Cailbhe Doherty

**Affiliations:** 1 School of Public Health, Physiotherapy and Sports Science, University College Dublin, Dublin 4, Ireland; 2 Insight Research Ireland Centre for Data Analytics, University College Dublin, Dublin 4, Ireland; 3 Institute for Sport and Health, University College Dublin, Dublin 4, Ireland; Università degli Studi di Milano: Universita degli Studi di Milano, ITALY

## Abstract

VO_2_ max is a measure of cardiorespiratory fitness and a key indicator of overall health. It is predictive of cardiovascular events and shows a strong inverse association with all-cause mortality. Increased cardiorespiratory fitness is associated with reductions in coronary artery disease, diabetes and cancer. Apple Watch offers a less resource-intensive and more feasible alternative to the gold standard assessment for VO_2_ max, indirect calorimetry, but the accuracy of its measurements remains uncertain. This study aimed to assess the validity of VO_2_ max estimates from Apple Watch in comparison to indirect calorimetry. Thirty participants wore an Apple Watch for 5-10 days to generate a VO_2_ max estimate. Subsequently, they underwent a maximal exercise treadmill test in accordance with the modified Åstrand protocol. The agreement between measurements from Apple Watch and indirect calorimetry was assessed using Bland-Altman analysis, mean absolute percentage error (MAPE), and mean absolute error (MAE). Overall, Apple Watch underestimated VO_2_ max, with a mean difference of 6.07 mL/kg/min (95% CI 3.77–8.38). Limits of agreement indicated variability between measurement methods (lower -6.11 mL/kg/min; upper 18.26 mL/kg/min). MAPE was calculated as 13.31% (95% CI 10.01–16.61), and MAE was 6.92 mL/kg/min (95% CI 4.89–8.94). These findings indicate that Apple Watch VO_2_ max estimates require further refinement prior to clinical implementation. However, further consideration of Apple Watch as an alternative to conventional VO_2_ max prediction from submaximal exercise is warranted, given its practical utility.

## Introduction

VO_2_ max, introduced by British physiologists Hill and Lupton, is the maximum amount of oxygen an individual can utilise during exercise [1]. It provides an evaluation of cardiorespiratory fitness and has been established as an important barometer of health [2–4]. Epidemiological evidence supports an inverse and independent association between cardiorespiratory fitness and mortality, as measured by VO_2_ max or metabolic equivalent (MET) [5–7]. It is a predictor of cardiovascular events [8–10], and some studies report it to be more predictive of cardiovascular and all-cause mortality than well-known risk factors such as hypertension, obesity, and hypercholesterolemia [11–13]. In recognition of its significance, the American Heart Association advocates for the routine assessment of cardiorespiratory fitness and has proposed it as a clinical vital sign [14].

VO_2_ max is the most widely accepted measure of cardiorespiratory fitness, and the gold standard for its assessment is indirect calorimetry [15]. Indirect calorimetry measures maximal oxygen uptake by recording oxygen consumption and carbon dioxide production during cardiopulmonary exercise testing (CPET) [16]. While it is the most accurate form of measurement, it is costly and resource-intensive, limiting its use in clinical practice and among the general population [17]. To overcome these difficulties, VO_2_ max is often derived from submaximal exercise tests that require less time and are considered lower-risk [18]. Error for VO_2_ max predictions from submaximal tests is reported to range between 1.6 and 4.1 mL/kg/min [19,20].

Wearable devices also use submaximal exercise to derive VO_2_ max [17], and their recent proliferation has democratised monitoring of physiological measurements. They provide unobtrusive, longitudinal monitoring outside the confines of exercise physiology laboratories or healthcare settings, and may facilitate remote monitoring of cardiorespiratory fitness [21,22]. The widespread uptake of wearable devices has been catalysed by the ready availability of a suite of health-related metrics, and their increasing prominence is illustrated by the American College of Sports Medicine (ACSM), who identified wearable technology as the number one fitness trend for 2025 [23]. Over half the population in many countries now owns a wearable device – ranging from smartwatches to smart rings – with global user numbers expected to grow significantly in the coming years [24]. The wearable technology market is projected to reach a valuation of $186 billion by 2030 [25], and Apple Watch holds the largest market share with over 100 million users worldwide [26].

The first-generation Apple Watch was released in 2015, and its VO_2_ max estimation feature was introduced with watchOS 4 in September 2017 [27]. This enabled Apple Watch Series 3 and later to estimate VO_2_ max using data such as heart rate, Global Navigation Satellite System-derived metrics (e.g., speed, distance, and elevation), and user demographics such as sex, age, height, and weight [28]. Apple subsequently updated its VO_2_ max algorithm in 2021 as part of watchOS 7 [28]. While this feature evoked the prospect of an assessment method that could be conducted independently of healthcare or exercise professionals, it was accompanied by notable challenges.

The accuracy of wearable-derived VO_2_ max estimates represents one of the most significant challenges. A multitude of factors can influence measurements: physiological factors such as heart rate response to exercise and perfusion; environmental factors like skin contact and motion [29]; or external influences, including caffeine intake and carrying additional weight beyond body weight, such as bags or equipment [28]. Compounding these issues is the proprietary and evolving nature of the algorithms used by wearable devices. With each new hardware or software update, algorithms are subject to change, necessitating recurrent validation by researchers [18,30,31]. This validation is hindered by the protracted process of conducting and publishing research. Consequently, fewer than 5% of consumer wearables have been validated for the full range of biometric outcomes they measure, including VO_2_ max [18]. In fact, just one study evaluating the accuracy of Apple Watch for VO_2_ max estimation has been published to date [32].

Evaluating the accuracy of VO_2_ max estimates is fundamental to integrating wearable devices into individual health monitoring and research contexts [30]. For consumers, the reliability and validity of these measurements can inform decisions about personal health and fitness. For researchers, wearables hold the potential to revolutionise public health surveillance by enabling the collection of large-scale, population-level data without the logistical and financial constraints of laboratory-based methods [33]. On the contrary, inaccurate or inconsistent data may compromise the validity of such research, particularly when used to inform health policy or population health interventions. Validation is required to seal the fissure between technological innovation and evidence-based application in health and fitness settings.

The aim of this study was to evaluate the accuracy of VO_2_ max estimates generated by Apple Watch in comparison to the gold standard method, indirect calorimetry. Specifically, the objectives were to assess the level of agreement between the two methods, and to quantify the degree of error in VO_2_ max estimates from Apple Watch.

## Materials and Methods

### Study design and oversight

This cross-sectional validation study was conducted in Dublin, Ireland between December 2023 and July 2024. Recruitment commenced on Monday, 4 December 2023, and finished on Monday, 22 July 2024. Healthy adults, aged 18 or older, were recruited through social media, posters, and word of mouth. Individuals with cardiovascular or mental illness, and those using medication affecting cardiovascular function were not eligible for inclusion. All participants completed the Physical Activity Readiness Questionnaire Plus (PAR-Q+) to ensure suitability for exercise, and written informed consent was obtained. Ethical approval was granted by the University College Dublin Human Research Ethics Committee (reference number: LS-23-55) on November 15th, 2023.

### Generating VO_2_ max estimates with Apple Watch

Apple Watch generates VO_2_ max estimates (proprietarily referred to as ‘Cardio Fitness’) by measuring an individual’s heart rate response to exercise during outdoor walking, running, or hiking activities on ground of less than 5% incline or decline [28]. Adequate GPS and heart rate signal must be obtained, alongside an increase of approximately 30% in heart rate from the resting value [28]. Multiple activities are required to generate an estimate, although this number varies between users [28]. Participants were informed of the required procedure and were requested to generate an estimate independently within one week of criterion testing. The process of generating a VO_2_ max estimate was conducted in accordance with manufacturer guidelines [28]. Participants were instructed to accurately input all required demographic information in the Health app prior to completing any exercise activities, including height, weight, sex, and age. If participants did not already own an Apple Watch, they were provided with an Apple Watch Series 9 or Ultra 2 for a period of 5-10 days. All Apple Watch devices were updated to watchOS 10 or later, ensuring that this validation study exclusively assessed devices using Apple’s latest VO_2_ max prediction algorithm and the most recent software version.

### Indirect calorimetry testing

Subsequently, each participant underwent a maximal cardiopulmonary exercise test (CPET) using indirect calorimetry at the Institute for Sport and Health, University College Dublin. Indirect calorimetry is considered the gold standard for VO_2_max testing [1,17]. An exercise treadmill test was conducted in accordance with the modified Åstrand protocol [34]. A treadmill speed between 8 and 13 km/h was selected and this remained consistent throughout [34]. The incline of the treadmill was increased by 2.5% every two minutes following the initial three-minute warm-up period at 0% incline [34]. The COSMED Quark CPET metabolic cart (COSMED, Trentino, Italy) was calibrated prior to each test according to manufacturer instructions and testing was conducted on the h/p/cosmos Venus treadmill (h/p/cosmos, Nußdorf-Traunstein, Germany). To ensure true VO_2_ max had been attained, participants were required to meet at least two of the following criteria: heart rate within ±10 bpm of age-predicted maximum (220 – age); respiratory exchange ratio (RER) ≥ 1.15; rate of perceived exertion (RPE) ≥ 17; VO_2_ plateau, defined as an increase in VO_2_ of less than 150 mL/min/kg, with an increase in work rate as evidenced by physiological data [35]. If at least two of these criteria were not met, the value was regarded as a VO_2_ peak. Participants were instructed to refrain from caffeine and nicotine for 12 hours prior to testing, and to avoid strenuous activity and alcohol for a minimum of 24 hours [15].

### Outcomes

The primary outcome was the agreement between VO_2_ max estimates derived from Apple Watch with those obtained via indirect calorimetry, which was calculated using Bland-Altman limits of agreement analysis [36], mean absolute percentage error (MAPE), and mean absolute error (MAE).

### Statistical analysis

The target sample size was calculated based on the sample sizes of previous studies investigating the measurement accuracy of other consumer wearable devices [32,37,38]. Additional participants were not recruited to account for potential dropouts due to the cross-sectional nature of the study design. Data from the COSMED Quark CPET were filtered using a time average of 30 seconds and exported to Microsoft Excel. The highest time-averaged VO_2_/kg value was interpreted as VO_2_ max. This was compared to the most recent Apple Watch VO_2_ max estimate available in the Health app. Participants who did not attain VO_2_ max were not included in the final analysis.

To evaluate the agreement between Apple Watch and the criterion, Bland-Altman limits of agreement analysis was conducted. The mean difference (bias) and 95% limits of agreement (LoA), defined as the mean difference ±1.96 times the standard deviation of the differences, were calculated. A Bland-Altman plot was generated to visually evaluate the agreement. Mean absolute percentage error (MAPE) and mean absolute error (MAE) were also calculated. The t-critical values were used to compute corresponding 95% confidence to account for the sample size. Analyses were conducted in Python (version 3.12) using pandas, Matplotlib, and NumPy packages. The scripts used are available at github.com/rorylambe/applewatch-validation.

## Results

### Baseline characteristics

A total of 30 individuals participated in this study (mean age [SD], 31.86 [13.99] years; 50% female). Of the 30 participants, two (one male, one female) did not achieve the required criteria for VO_2_ max, and as such 28 participants were included in the analyses. One of the excluded participants met only the threshold for rate of perceived exertion whereas the other failed to attain any of the criteria. Both excluded participants were wearing Apple Watch Series 9. The cardiorespiratory fitness level of each participant, determined by indirect calorimetry testing, was classified in accordance with the reference standard from the Fitness Registry and the Importance of Exercise National Database (FRIEND) [39]. 21 participants were classified as having either ‘Superior’ or ‘Excellent’ cardiorespiratory fitness, while nine individuals were classified as ‘Good’ or ‘Fair’. Participant baseline characteristics, including BMI and Fitzpatrick skin tone, are listed in Table 1.

**Table 1:** Characteristics of the participants at baseline.

Characteristic	Value
Age, mean (SD), years	31.86 (13.99)
Female, no. (%)	15 (50%)
Height, mean (SD), cm	172.23 (7.85)
Weight, mean (SD), kg	70.55 (9.13)
BMI, mean (SD), kg/m^2^	23.76 (2.54)
Fitzpatrick skin tone ^a^	
Type I	4
Type II	22
Type III	4
CRF level ^b^	
Superior, no.	3
Excellent, no.	18
Good, no.	8
Fair, no.	1
Apple Watch model ^c^	
Series 9	17
Ultra 2	6
Series 8 and SE 2	2 of each
Series 5, 6, 7	1 of each

BMI: Body Mass Index

^a^ Number of participants of each skin tone according to the Fitzpatrick Scale.

^b^ Number of participants of each cardiorespiratory fitness level classified according to FRIEND.

^c^ Number of participants who wore each Apple Watch model during the study.

### Apple Watch agreement with the criterion

**Fig 1:**
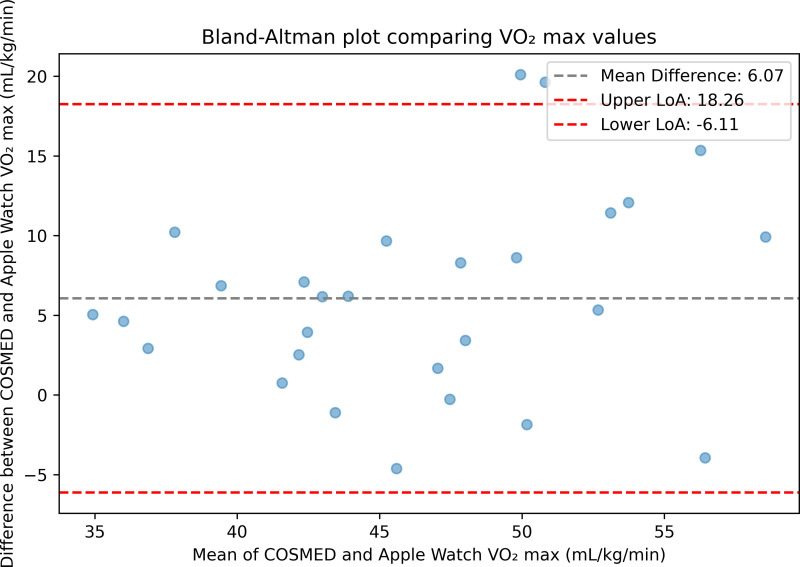
Bland-Altman plot demonstrating the agreement between VO2 max measurements from Apple Watch and the criterion measure. Upper LoA = upper limit of agreement; Lower LoA = lower limit of agreement.

Overall, Apple Watch underestimated VO_2_ max in comparison to indirect calorimetry. The mean difference was 6.07 mL/kg/min (SD 6.22; 95% confidence interval [CI] 3.77–8.38). Bland-Altman limits of agreement (LoA) indicated variability between the two measurement methods (lower LoA -6.11 mL/kg/min; upper LoA 18.26 mL/kg/min). The Bland-Altman plot is presented in Fig 1. The mean absolute percentage error (MAPE) was 13.31% (95% CI 10.01–16.61); mean absolute error (MAE) was 6.92 mL/kg/min (95% CI 4.89–8.94). To assess statistical power and effect size, a one‐sample t‐test was conducted comparing Apple Watch and criterion VO_2_ max values. This analysis yielded a t‐value of –5.07 (df = 27, p < 0.001), indicating a significant difference with a large effect size (Cohen’s d = –0.96). A post-hoc power analysis (α = 0.05) confirmed the sample size (n = 28) provided >99% power, suggesting the study was sufficiently powered to detect a true difference between the measurement methods, thereby minimising the risk of Type II error. No trends in accuracy could be observed based on Apple Watch model. Results are summarised in Table 2.

**Table 2:** Statistical measures of agreement between Apple Watch and indirect calorimetry.

**Statistical measure**	**Result**
Mean (SD), mL/kg/min	
Apple Watch	43.27 (6.34)
COSMED*	49.35 (7.79)
Standard error of the mean, mL/kg/min	
Apple Watch	1.20
COSMED	1.47
Standard deviation of the differences, mL/kg/min	6.22
Mean difference (95% CI), mL/kg/min	6.07 (3.77–8.38)
Bland-Altman limits of agreement (lower, upper), mL/kg/min	−6.11, 18.26
Mean absolute percentage error (95% CI)	13.31% (10.01–16.61)
Mean absolute error, mL/kg/min (95% CI)	6.92 (4.89–8.94)

* COSMED = COSMED Quark CPET metabolic cart.

## Discussion

This validation study evaluated the accuracy of VO_2_ max estimates from Apple Watch compared to the gold-standard method of indirect calorimetry. Overall, Apple Watch underestimated VO_2_ max, with a mean difference of 6.07 mL/kg/min (SD 6.22) and a MAPE of 13.31%. Bland-Altman limits of agreement indicated variability between the two measurement methods (lower -6.11 mL/kg/min; upper 18.26 mL/kg/min).

These findings align with those of a prior study evaluating Apple Watch Series 7, which reported a similar mean underestimation of 4.5 mL/kg/min [32]. However, important methodological differences exist. In the previous study, criterion VO_2_ max values were obtained through a graded exercise test conducted on a cycle ergometer, rather than using a treadmill-based test. Apple Watch derives VO_2_ max from walking, running or hiking activities [28], and comparison of VO_2_ max measured through different exercise modalities – such as cycling compared with running – is not advised due to the inherent discrepancies in results they produce [40]. Typically, treadmill tests yield higher VO_2_ max values, although the difference is smaller in well-trained cyclists [41]. This difference underpins the INTERLIVE consortium’s recommendation that criterion testing methods should closely align with the activity used by the wearable device to generate VO_2_ max predictions [17].

While these methodological differences are important to consider, the authors noted distinct trends based on the fitness levels of participants [32]. For individuals with good or excellent fitness, Apple Watch demonstrated a propensity to underestimate VO_2_ max, whereas among those with poor fitness, there was a tendency to overestimate. Although these authors found an overall underestimation, a separate meta-analysis of fourteen studies, which included multiple wearable device brands, reported a minor overestimation (pooled bias -0.09 mL/kg/min) [17]. Despite a very low bias, the limits of agreement were comparatively wide (lower -9.92 mL/kg/min; upper 9.74 mL/kg/min), suggesting both positive and negative differences in estimates of a relatively large magnitude, which collectively yielded a bias close to zero. A propensity to both under- and overestimate may arise from prediction algorithms that are developed using population-level data or mean values for specific demographics. Given our study sample predominantly consisted of individuals with high cardiorespiratory fitness levels, this may constitute one explanation for our finding of underestimation. Importantly, it highlights the need to consider participant demographic characteristics, as well as multiple statistical measures of agreement, when interpreting results.

Our findings, combined with the broader literature, underscore the challenge of deriving maximal oxygen consumption from submaximal exercise for wearable devices. Like conventional prediction equations, Apple’s prediction algorithm is founded on heart rate response to exercise [28]. The inclusion of machine learning techniques and additional motion sensor measurements is also probable, although the precise nature of the algorithm remains undisclosed [30]. In clinical settings, submaximal exercise testing is often used alongside prediction equations to estimate VO_2_ max, owing to safety and feasibility concerns [35]. These equations predict VO_2_ max based on an individual’s physiological response to exercise of a quantified workload, and six assumptions – outlined by the ACSM – must be achieved to ensure optimal accuracy [35]. They include a minimal difference between actual and predicted maximal heart rate, knowledge of medication or substances affecting heart rate, and the existence of a linear relationship between heart rate and work rate. Yet, accuracy from traditional prediction equations can vary significantly [42–44]. A study (n = 541) evaluating three different submaximal exercise tests for estimating maximal oxygen uptake reported a standard error of the estimation ranging from 3.7 to 4.5 mL/kg/min [45]. In a separate study examining the Chester Step Test, the predicted aerobic capacity had a standard error of 3.9 mL/kg/min [19,46]. Comparatively, measurement error for laboratory-based indirect calorimetry has been estimated at ±5%, with a meta-analysis of 39 studies reporting a mean standard error of 2.58 mL/kg/min [40,47]. Considering this literature, the results of our validation study indicate that Apple Watch VO_2_ max predictions fall approximately two to three standard deviations beyond the typical error of the gold standard measurement. However, they align more closely with the magnitude of error reported for traditional VO_2_ max predictions derived from submaximal exercise.

While machine learning and continuous monitoring are distinct merits of Apple Watch compared to traditional prediction methods, the uncontrolled nature of the exercise used to generate Apple Watch VO_2_ max estimates presents a considerable challenge. Limited information of external factors that affect heart rate response to exercise – including exercise surface, environmental temperature, or alcohol intake – can adversely impact predictions [28]. Moreover, heart rate response to lower-intensity exercise – often used to estimate VO_2_ max – varies considerably between individuals [48]. Estimates based on such exercise may misinform predictions. Due to the proprietary nature of Apple’s algorithm, we remain unaware of the specific data, or features, used to train and develop the prediction model. Therefore, we cannot be certain what factors most influence accuracy, or in which instances predictions are most reliable [30]. Considering this, a defined testing protocol – requiring users to input specific information and conduct a particular type of exercise – may reduce the level of uncertainty and error. Despite the imprecision of predictions from submaximal exercise – both from traditional testing methods and wearable devices – its widespread clinical use illustrates its value. The potential for Apple Watch to provide a commensurate assessment method that can be conducted independently by members of the general public warrants careful consideration. To investigate this, validation of contemporary technology is apt.

Continual, or ‘living’, validation is essential to keep pace with the iterative commercial ecosystem. The annual update cycle of Apple Watch poses a significant challenge to evaluating current technology, and the prolonged academic publication process often means that the hardware or software under validation has been discontinued by the time of publication [18,30]. This is accentuated by the rapid advancement of machine learning – and the associated updates to watchOS – which play an increasingly important role in providing accurate measurements due to the complexity of interpreting multiple sensor measurements and sensitive photoplethysmography waveforms [49,50]. Amidst these challenges, this study provides a timely evaluation of Apple’s most recent optical heart rate sensor and its latest VO_2_ max prediction algorithm [28,51]. It is the first study to evaluate Apple Watch Series 9 and Ultra 2. As wearable technology evolves, agile validation will be critical to realising the potential of consumer wearables in large-scale public health applications and personalised fitness monitoring.

Central to this potential is the self-directed nature of wearable device measurements, and this study’s methodology was designed to reflect this. Participants were not required to adhere to a prescribed protocol to generate a VO_2_ max prediction. Rather, they were solely informed of the requirements, meaning that estimates were generated through activities that more accurately reflected each individual’s real-world use, enhancing the ecological validity of our findings. Additionally, a robust statistical approach was used, guided by recommendations of the INTERLIVE expert consortium [17], and a sex-balanced study sample was recruited. However, the predominance of participants with high levels of cardiorespiratory fitness represents the study’s most significant limitation. Only one participant was classified as having ‘Fair’ cardiorespiratory fitness, while all others were categorised as ‘Good’, ‘Excellent’, or ‘Superior’. This limits the generalisability of our findings to populations with lower fitness levels, including clinical cohorts. Additionally, the sample primarily consisted of younger individuals, despite efforts to recruit participants across a broad age range. Another limitation is the uncontrolled procedure of generating Apple Watch VO_2_ max estimates. While this protocol reflects real-world use, it introduces unquantified external variables that may have influenced prediction accuracy. Lastly, only one VO_2_ max estimate was collected per participant, preventing analysis of intra-subject variability, which may have provided insights into reliability and changes in measurement accuracy over time.

The findings of this study have important implications for the use of wearable devices such as Apple Watch in clinical and research contexts. VO_2_ max estimates from Apple Watch offer a cost-effective and scalable alternative to laboratory-based testing, enabling studies involving diverse populations, as well as digital endpoints for clinical trials. However, given the observed variability and underestimation of VO_2_ max in this study, caution is warranted when interpreting the data in clinical or research settings. While Apple Watch may provide a suitable estimation of aerobic capacity for general fitness monitoring, our findings suggest that estimates are not sufficiently accurate to inform clinical decision-making. Future research should include individuals with diverse cardiorespiratory fitness levels to enhance generalisability, support the refinement of predictive algorithms, and develop frameworks for ongoing evaluation of wearable devices. Such efforts are essential to bridging the gap between technological innovation and evidence-based practice, ultimately enhancing the utility of wearable devices for individual health monitoring and public health applications.

## Conclusions

This study found that Apple Watch underestimated VO_2_ max compared to indirect calorimetry in a sample predominantly composed of individuals with high cardiorespiratory fitness levels. The margin of error more closely aligned with that of VO_2_ max derived from conventional submaximal exercise testing than with VO_2_ max obtained via indirect calorimetry. The unstructured nature of the exercise used to generate Apple Watch VO_2_ max predictions introduces external factors – such as environmental conditions and variations in heart rate response to exercise – that are not fully accounted for by the device. Nevertheless, Apple Watch holds promise as a practical and accessible alternative to conventional submaximal exercise testing. Its potential clinical utility warrants further investigation, and ongoing validation is required to evaluate its accuracy as hardware and software evolve. Future research should focus on expanding validation efforts to include diverse populations, ensuring these devices can be effectively integrated into both individual health monitoring and broader public health applications.
