# Supplementary material for: Investigating the accuracy of Apple Watch VO2 max measurements: A validation study
Source: PLoS One. 2025 May 15;20(5):e0323741. doi: 10.1371/journal.pone.0323741 (PMC12080799; doi:10.1371/journal.pone.0323741)
Supplement: Attachment — Submitted filename: Response to Reviewers.docx [file pone.0323741.s002.docx]

**Response to Reviewers**

**Response to Academic Editor**

**COMMENT:** 1. Please ensure that your manuscript meets PLOS ONE's style requirements, including those for file naming. The PLOS ONE style templates can be found at <https://journals.plos.org/plosone/s/file?id=wjVg/PLOSOne_formatting_sample_main_body.pdf> and <https://journals.plos.org/plosone/s/file?id=ba62/PLOSOne_formatting_sample_title_authors_affiliations.pdf>

**RESPONSE:** Font size for headings and subheadings has been altered, in accordance with style and formatting requirements. A caption has been inserted for Figure 1 — please note that Fig 1 in this submission is the figure that was named Figure 2 in the previous draft. Figure 1 from the previous submission has been removed in response to the comments of Reviewer 2.

Sentence case has been implemented for the title, and the Acknowledgements section has been reformatted. File names have also been altered.

--

**COMMENT:** 2. Your ethics statement should only appear in the Methods section of your manuscript. If your ethics statement is written in any section besides the Methods, please delete it from any other section.

**RESPONSE:** The ethics statement has been removed from the Acknowledgements section, and now only appears in the Methods section.

--

**COMMENT:** 3. Please ensure that you refer to Figure 2 in your text as, if accepted, production will need this reference to link the reader to the figure.

**RESPONSE:** In this submission, there is now just one figure — in response to the comments of Reviewer 2. A reference to Figure 1 has been included in the text (line 254, under sub-heading ‘Apple Watch agreement with the criterion’):

*“The Bland-Altman plot is presented in Fig 1.”*

--

**Response to Reviewer 1**

**COMMENT:** The study compares the accuracy of Apple Watch VO₂ max predictions to indirect calorimetry, in order to address a crucial problem in wearable health technologies. The topic is extremely pertinent in light of the growing use of consumer wearable technology for health monitoring. The backdrop is organized effectively, highlighting the significance of VO₂ max as a health indicator and recognizing the shortcomings of the available evaluation techniques.

**RESPONSE:** Thank you for your constructive comments highlighting the importance of this topic and its growing pertinence. I’m grateful for your feedback as it has helped us to improve this paper.

**COMMENT:** The testing procedure, exclusion criteria, and participant recruiting are all covered in depth in the methodology's extensive documentation. But there are a few issues:

- The sample size (n=30, of which 28 were analyzed) is too small to extrapolate results.

- The study's relevance to populations with lower fitness levels may be limited because it mostly involves people with strong cardiorespiratory fitness.

**RESPONSE:** Thank you for recognising the detail of this manuscript’s methodology. We certainly agree that the size of the sample — and the predominance of individuals with high level of cardiorespiratory fitness — limits the extrapolation of our results to the general population. A larger sample size including individuals with a wide range of cardiorespiratory fitness would greatly enhance the generalisability of our findings, and we acknowledge this as an important limitation in the Discussion section of the manuscript.

We have now conducted a post-hoc power analysis for our sample size, using the following information from our results:

- Apple Watch mean VO_2_ max (standard deviation): 43.27 (6.34)
- Criterion mean VO_2_ max: 49.35
- Sample size: 28

We calculated the following results from the post-hoc power analysis:

- Standard Error: 1.198
- t value: -5.075
- p-value: 0.00002
- Cohen's d: -0.959
- Power: 0.998

The results of this power analysis suggest our study is well-powered to detect an effect. We have now added this analysis to the Results section of our manuscript:

“To assess statistical power and effect size, a one‐sample t‐test was conducted comparing Apple Watch and criterion VO₂ max values. This analysis yielded a t‐value of –5.07 (df = 27, p < 0.001), indicating a significant difference with a large effect size (Cohen’s d = –0.96). A post-hoc power analysis (α = 0.05) confirmed the sample size (n = 28) provided >99% power, suggesting the study was sufficiently powered to detect a true difference between the measurement methods, thereby minimising the risk of Type II error.”

While a concerted effort was made by our research team to recruit individuals across a wide range of age and fitness levels, this recruitment proved challenging. Due to the exhaustive nature of a maximal exercise test, few individuals with low levels of cardiorespiratory fitness levels were willing to participate, and all included participants aged 55-65 had ‘Excellent’ or ‘Superior’ cardiorespiratory fitness. Previously, just one study evaluating the accuracy of VO_2_ max measurements from Apple Watch has been published. That study compared predictions from Apple Watch Series 7 to VO_2_ max values obtained during a cycling protocol, measured using a portable gas analyser (n=19, 7 female). This study builds on the current literature base by providing the first assessment of Apple Watch Series 9 and Ultra 2, in a larger sex-balanced cohort. This is the first study to compare Apple Watch VO_2_ max measurements to indirect calorimetry — regarded as the criterion standard — which were obtained during an exercise treadmill test, closely mirroring the activity required to generate an Apple Watch prediction. This is in accordance with guidelines for validation published by the expert-led INTERLIVE consortium.

--

**COMMENT:** There is no explicit explanation in the study as to why particular Apple Watch models were chosen over others or whether hardware variations affected the findings.

**RESPONSE:** Thank you for highlighting this important point. At the time of data collection, the most recently released models were Apple Watch Series 9 and Ultra 2. The Series 9 has recently been replaced by Series 10, while the Ultra 2 remains the flagship model. A substantial number of these devices were purchased to provide to participants (17 x €449 for Series 9, 6 x €899 for Ultra 2) so that the latest device could be validated. Due to funding restraints, and in an effort to recruit a greater number of participants, the authorship team also recruited individuals who owned an Apple Watch which was capable of running watchOS 10 or later. In this way, all models included in this validation study were running the latest version of watchOS software, and all devices used the latest algorithm to generate a VO_2_ max prediction.

The following information has been added in the Methods section, under the sub-heading ‘Generating VO_2_ max estimates with Apple Watch’:

*“If participants did not already own an Apple Watch, they were provided with an Apple Watch Series 9 or Ultra 2 for a period of 5-10 days to generate an estimate. All Apple Watch devices were updated to watchOS 10 or later, ensuring that this validation study exclusively assessed devices using Apple’s latest VO_2_ max prediction algorithm and the most recent software version.”*

No trends in accuracy could be observed based on the individual Apple Watch model tested, nor based on participant demographic (please also see our response to the following comments). This may indicate that the prediction algorithm has a large role in determining the accuracy of VO_2_ max estimates, compared to differences in hardware. To facilitate transparent reporting of results, we have uploaded additional data relating to Apple Watch model and participant demographic to github.com/rorylambe/applewatch-validation.

We have also added additional information about hardware variations to the Results section of the manuscript.

*--*

**COMMENT:** The statistical approach is relative good, employing Bland-Altman analysis, mean absolute percentage error (MAPE), and mean absolute error (MAE) to assess agreement. However, the study could have benefited from additional analysis:

- A regression model to explore factors influencing the discrepancies.
- Subgroup analysis to determine whether the accuracy of estimates varies by demographic factors (e.g., age, sex, fitness level).

**RESPONSE:** This was a very thought-provoking comment, and the authorship team spent much time examining possibilities for further analyses. We designed the statistical analysis plan of this study in accordance with the expert-led INTERLIVE consortium’s recommendations. To explore the potential influence of these factors, we have conducted two linear regression models — one each for age, and sex. Regression was chosen as a statistically robust approach that adjusts for cofounders and retains sample size.

To reduce a risk of overfitting and to maximise statistical power, we chose to run separate simple linear regression models for sex and age rather than a multiple regression model. Despite this, both models remain statistically underpowered and prone to collinearity. As a consequence, having conducted these regression analyses, we feel that it may be more suitable not to include these underpowered analyses in the final manuscript. Additional analyses were not part of the initial statistical analysis plan, and the authorship team do not believe that sex or age is likely to have a substantial influence given the nature of the cohort recruited by Apple in their development of their proprietary algorithm.

Neither regression models revealed a statistically significant association. For sex, we found that the level of agreement between Apple Watch and the criterion measurement was greater for males, although this was not statistically significant. Sex accounted for 12.7% of the variance in discrepancy between measurement methods. Age did not demonstrate a statistically significant association, and it accounted for just 2% of variance.

Although a regression model for fitness level would be of substantial value, we determined that it would be inappropriate to conduct in this instance. This is due to the nature of the spread of cardiorespiratory fitness levels across each category, and the resulting number of individuals per category. There is an inadequate number of participants in each category for sufficient statistical power, and to account for collinearity.

We have, however, prepared an updated version of the Results sections of the manuscript to include if the Academic Editor deems these regression models to be of additional value to the paper.

Results:

Overall, Apple Watch underestimated VO2 max in comparison to indirect calorimetry. The mean difference was 6.07 mL/kg/min (SD 6.22; 95% confidence interval [CI] 3.77–8.38). Bland-Altman limits of agreement (LoA) indicated variability between the two measurement methods (lower LoA -6.11 mL/kg/min; upper LoA 18.26 mL/kg/min). The Bland-Altman plot is presented in Fig 1. The mean average percentage error (MAPE) was 13.31% (95% CI 10.01–16.61); mean absolute error (MAE) was 6.92 mL/kg/min (95% CI 4.89–8.94). To assess statistical power and effect size, a one‐sample t‐test was conducted comparing Apple Watch and criterion VO₂ max values. This analysis yielded a t‐value of –5.07 (df = 27, p < 0.001), indicating a significant difference with a large effect size (Cohen’s d = –0.96). A post-hoc power analysis (α = 0.05) confirmed the sample size (n = 28) provided >99% power, suggesting the study was sufficiently powered to detect a true difference between the measurement methods, thereby minimising the risk of Type II error. No trends in accuracy could be observed based on Apple Watch model. Results are summarised in Table 2. *“Two separate linear regression models were used to examine the associations of sex and age (predictor variables in respective models), with the signed difference in VO_2_ max values between Apple Watch and the criterion (dependent variable in each model). Neither sex nor age demonstrated a statistically significant association with measurement accuracy. Sex accounted for 12.7% of the variance in discrepancy between measurement methods (β = -4.359, SE = 2.237; t = -1.949; 95% CI: -8.956–0.239; R2 = 0.127; F = 3.797). The coefficient for male sex indicated that the level of agreement was, on average, 4.36 mL/kg/min higher in males compared to females, however, this was not statistically significant (p = 0.062). Age explained just 2% of variance and was not a significant predictor (β = 0.0623, SE = 0.085; t = 0.734; 95% CI: -0.112–0.237; R2 = 0.02; F = 0.539). It was deemed inappropriate to conduct regression based on cardiorespiratory fitness due to the imbalanced spread of participants across each category. Additionally, no trends in accuracy could be observed based on Apple Watch model. Raw results data, along with demographic information used for regression, has been published at github.com/rorylambe/applewatch-validation.”*

--

**COMMENT:** The authors appropriately cite relevant literature, including a meta-analysis on wearable-derived VO₂ max estimates. They also highlight the proprietary nature of Apple’s prediction algorithm as a limitation, a crucial consideration in wearable validation research. The study suggests that Apple Watch could serve as an alternative to traditional submaximal exercise testing but requires further refinement. However, the discussion could be improved by:

- Addressing whether firmware updates might influence VO₂ max accuracy over time.

**RESPONSE:** In response to your comments, we have included your suggestions in the Discussion.

Hardware and software both influence the accuracy of VO_2_ max estimates. Advancements in photoplethysmography sensors — in particular increases in light frequency and intensity — have yielded improvements in heart rate accuracy, during high-intensity activities especially. These heart rate measurements form a critical element of prediction algorithms for VO_2_ max. A growing source of improvement in the accuracy of these predictions, however, is the machine learning (ML) that underpins prediction algorithms. Rapid advancements in ML are facilitating improved recognition of motion artefacts, and detection of important segments in photoplethysmography waveforms. Consequently, firmware updates will have a substantial impact on accuracy, however, significant updates can only be achieved by the development of new machine learning algorithms which requires the collection of substantial amounts of data. This is one reason we do not see yearly updates to Apple’s VO_2_ max prediction algorithm.

We have added the following information to the Discussion:

“The annual update cycle of Apple Watch poses a significant challenge to evaluating current technology, and the prolonged academic publication process often means that the hardware or software under validation has been discontinued by the time of publication [18, 28]. *This is accentuated by the rapid advancement of machine learning — and the associated updates to watchOS — which play an increasingly important role in providing accurate measurements due to the complexity of interpreting multiple sensor measurements and sensitive photoplethysmography waveforms [43, 44*]. Amidst these challenges, this study provides a timely evaluation of Apple’s most recent optical heart rate sensor and its latest VO₂ max prediction algorithm [26, 45].”

--

**COMMENT:** Providing a clearer stance on whether Apple Watch is suitable for specific use cases (e.g., general fitness tracking vs. clinical decision-making).

**RESPONSE:** We acknowledge the importance of providing a clear interpretation of our results, and their implications for use cases. To address your comment, we have added additional information to the Discussion. In doing so, we aim to clarify that Apple Watch VO_2_ max predictions do not demonstrate sufficient accuracy to inform clinical decisions, based on our findings. Although estimates are accompanied by a degree of inaccuracy, Apple Watch may provide an accessible estimation of aerobic capacity for the purpose of general fitness monitoring.

The following amendments have been made:

“VO₂ max estimates from Apple Watch offer a cost-effective and scalable alternative to traditional laboratory-based testing, enabling studies involving diverse populations. However, given the observed variability and underestimation of VO₂ max in this study, caution is warranted when interpreting the data in clinical or research settings. While Apple Watch may provide a suitable estimation of aerobic capacity for general fitness monitoring, our findings suggest that estimates are not sufficiently accurate to inform clinical decision-making.”

--

**COMMENT:** While the study contributes valuable insights, its limitations—small sample size, fitness level bias, and high variability in agreement—raise concerns about its broader applicability.

With above suggestions, the paper would be more robust and suitable for publication.

**RESPONSE:** The research team is very grateful for your comments on this manuscript because they facilitated a more comprehensive scientific paper. We aimed to implement your suggestions to ensure a more robust evaluation and description of our study. We conducted a post-hoc power calculation, two regression models, and have implemented suggested edits to the Discussion section.

--

**Response to Reviewer 2**

**COMMENT:** I would like to applaud the authors on this work, it is important to scrutinize the quality of data coming out of "consumer devices" and we need more research focus on this topic. I only have a few suggestions for the authors, some I would consider "major" and would need to be addressed for publication and some I would consider optional. Overall, I think the manuscript can be improved with minimal effort and will be suitable for publication.

**RESPONSE:** We greatly appreciate your comments and acknowledgement of the pertinence of the research topic. We have found your feedback to be very beneficial for this manuscript, and we hope that we adequately address each of your suggestions.

--

**COMMENT:**

Major suggestions:

(1) Fix your figures. I see no purpose for Fig 1 as it stands, the data from it is actually in Fig 2 and the graphics are neither discussed nor referenced. Fig 2 is also not referenced anywhere in the manuscript. And please add some captions. The figures are probably the weakest part of this manuscript.

**RESPONSE:** We acknowledge that the data in Fig 1 is also provided in the text of the Results section, as well as in the Bland-Altman plot. The initial purpose of Fig 1 was to provide the reader with an accessible, and easily interpretable, summary of findings. However, in response to your comment, we have removed this figure.

Figure 1 is now the Bland-Altman plot. We have included reference to the figure in the Results section of the manuscript and we have also added captions:

**Fig 1. Bland-Altman plot demonstrating the agreement between VO2 max measurements from Apple Watch and the criterion measure.** Upper LoA = upper limit of agreement; Lower LoA = lower limit of agreement.

--

**COMMENT:** (2) Bring some data / discussion into the manuscript around the repeatability and/or within-subject variance of the gold standard test. It would have been great if the authors collected more than 1 VO2 MAX estimate / subject to be able to derive this from their own data, however, I would be happy with just reference values from published literature. And then the authors should compare the Apple Watch bias / precision / accuracy to the variance of the gold standard test. For example, I would really want to know: is 6 mL/kg/min difference within 1,2,3 SDs of the gold standard test?

**RESPONSE:** This is a very good observation. It has added great value to the manuscript. We gladly examined the available literature for studies assessing the reliability of laboratory-based indirect calorimetry using a metabolic cart. We also explored research on prediction equations which are used in conjunction with submaximal exercise tests.

Despite its widespread use, there are comparatively few high-quality studies with large cohorts that have assessed the within-subject variance for the COSMED Quark metabolic cart. Nonetheless, the authorship team identified a number of studies. Based on the findings of one meta-analysis – and a review article which included multiple studies – we found that the measurement error for indirect calorimetry has been reported as approximately ±5%, or 2.58 mL/kg/min.

For maximal oxygen uptake predictions based on submaximal exercise tests, the margin of error is larger, and more comparable to our findings for Apple Watch. One large study reported a range of 3.7 to 4.5 mL/kg/min for the standard error of the estimation.

We have included this additional data in the Discussion and have compared our findings to the variance reported for maximal and submaximal testing. To accommodate this important additional information, we have also made some alterations to the structure of the Discussion to improve flow and readability.

The following was added:

“These include a minimal difference between actual and predicted maximal heart rate, knowledge of medication or substances affecting heart rate, and the existence of a linear relationship between heart rate and work rate. Yet, accuracy from traditional prediction equations can vary significantly [42-44]. A study (n = 541) evaluating three different exercise tests for estimating maximal oxygen uptake reported a standard error of the estimation ranging from 3.7 to 4.5 mL/kg/min [45]. In a separate study examining the Chester step test, the predicted aerobic capacity had a standard error of 3.9 mL/kg/min [19, 46]. Comparatively, measurement error for laboratory-based indirect calorimetry has been estimated at ±5%, with a meta-analysis of 39 studies reporting a mean standard error of 2.58 mL/kg/min [40, 47]. Considering this literature, the results of our validation study indicate that Apple Watch VO_2_ max predictions fall approximately two to three standard deviations beyond the typical error of the gold standard measurement. However, they align more closely with the magnitude of error reported for traditional VO_2_ max predictions derived from submaximal exercise.”

--

**COMMENT:** (3) Include all key parameters that Apple uses in their algo. For example, "sex" and "height" are missing on page 4, line 84 and "sex" is missing on page 6, line 140.

**RESPONSE:** Thank you for this observation. We have made the following amendments at the specified parts of the manuscript text:

“This enabled Apple Watch Series 3 and later to estimate users' VO₂ max using data such as heart rate, Global Navigation Satellite System-derived metrics (e.g., speed, distance, and elevation), *and user demographics, including sex, age, height, and weight* [26]. Apple updated its VO₂ max algorithm in 2021 as part of watchOS 7 [26].”

“Participants were instructed to accurately input all required demographic information in the Health app prior to completing any exercise activities, including *height, weight, sex, and age.*”

--

**COMMENT:** (4) Page 7, in the "Outcomes" section of the Methods, provide a reference to the Bland and Altman work, for example: Altman DG, Bland JM. Measurement in medicine: the analysis of method comparison studies. Statistician. 1983;32:307–17. 10.2307/2987937 or Bland JM, Altman DG. Statistical methods for assessing agreement between two methods of clinical measurement. Int J Nurs Stud. 2010;47:931–6. 10.1016/j.ijnurstu.2009.10.001

**RESPONSE:** Thank you. We have now added a reference to their work in this section.

--

**COMMENT:**
Minor suggestions:

(1) Include some data from other submaximal exercise tests (mentioned on page 3, second paragraph) and discuss how other tests compare to the Apple Watch estimate.

**RESPONSE:** We have included additional data on other submaximal exercise tests in the introduction to provide improved context of measurement accuracy at the beginning of the manuscript:

“Error for VO_2_ max predictions from submaximal tests are reported to range between 1.6 and 4.1 mL/kg/min [19, 20].”

We also discuss traditional submaximal exercise tests – and their comparison with Apple Watch-derived predictions – in more detail in the Discussion section of the manuscript.

--

**COMMENT:** (2) Calculate not only the bias difference across testing methods but also the precision and create BA plots for those too.

**RESPONSE:** Thank you for this suggestion. In our study, we designed our statistical analysis plan based on the expert‐led INTERLIVE consortium’s recommendations for statistical analysis of wearable-derived VO₂ max validation, which primarily focus on assessing bias and agreement via Bland–Altman plots.

These guidelines recommend Bland-Altman analysis with limits of agreement, and a plot for visual inspection of results, as well as computing Mean Average Percentage Error. As we only collected one Apple Watch measurement per participant, we were unable to evaluate precision within the Apple Watch measurements, a limitation that we acknowledge. We recognise that including precision analyses alongside bias differences would offer additional insights, and we appreciate this recommendation for future research designs.

--
