# Supplementary material for: Investigating the accuracy of Apple Watch VO2 max measurements: A validation study
Source: PLoS One. 2025 May 15;20(5):e0323741. doi: 10.1371/journal.pone.0323741 (PMC12080799; doi:10.1371/journal.pone.0323741)
Supplement: Attachment — Submitted filename: Response_to_Reviewers_auresp_2.docx [file pone.0323741.s003.docx]

**Response to Reviewers**

**Journal Requirements:**

**COMMENT:** Please review your reference list to ensure that it is complete and correct. If you have cited papers that have been retracted, please include the rationale for doing so in the manuscript text, or remove these references and replace them with relevant current references. Any changes to the reference list should be mentioned in the rebuttal letter that accompanies your revised manuscript. If you need to cite a retracted article, indicate the article’s retracted status in the References list and also include a citation and full reference for the retraction notice.

**RESPONSE:** Each of the 52 references have now been cross-checked by a hand-search to ensure that they are correct. Alterations have been made to the following references:

Hill and Lupton (reference number 1), Grand View Research (reference number 25), Statista wearable device ownership (reference 24), Apple watchOS (reference 27), Apple Cardio Fitness estimates (reference 28), ACSM fitness trends (reference 23), Astrand protocol (reference 35).

No retracted papers have been cited.

--

**Reviewer 1:**

**COMMENT:** The revised manuscript presents a well-structured and thorough validation study on the accuracy of Apple Watch VO₂ max estimates compared to the gold-standard method of indirect calorimetry. The authors have effectively addressed the concerns raised in the initial review by improving methodological transparency, incorporating additional statistical analyses, and expanding the discussion to provide better context for their findings. The inclusion of a post-hoc power analysis strengthens the study’s credibility, confirming that the sample size of 28 participants was statistically sufficient to detect meaningful differences between Apple Watch and indirect calorimetry. Additionally, the authors conducted regression analyses to assess the influence of age and sex on measurement discrepancies, although no significant associations were found. These additions enhance the depth of the study and demonstrate a rigorous approach to validation.

A major improvement in the revised version is the expanded discussion, which now situates Apple Watch’s performance within the broader context of wearable technology and traditional submaximal exercise tests. The authors acknowledge the proprietary nature of Apple’s algorithm and how software updates may influence accuracy over time. Furthermore, they compare the observed error margins to those of conventional submaximal VO₂ max prediction methods, reinforcing the notion that Apple Watch may be more suitable for general fitness tracking than for clinical decision-making. The discussion also now includes a reference to the inherent variability of indirect calorimetry (~2.58 mL/kg/min), providing a useful benchmark for interpreting the Apple Watch’s underestimation of VO₂ max by 6.07 mL/kg/min.

**RESPONSE:** We are very grateful for your valuable comments during the previous round of review. Your review allowed us to greatly strengthen the manuscript. We conducted post-hoc power analysis, as well as regression analyses, and expanded the Discussion section. Additionally, the comparison of Apple Watch’s error margin to that of conventional testing adds substantially to the interpretation of our results — thank you for this recommendation.

--

Despite these strengths, some limitations remain. The sample is still skewed towards individuals with high cardiorespiratory fitness, which restricts the generalizability of findings to less fit or clinical populations. Although the authors acknowledge this in the discussion, future studies should aim for a more diverse participant pool. Another limitation is the uncontrolled nature of the Apple Watch data collection process, as participants generated VO₂ max estimates independently over several days without standardized exercise conditions. While this enhances ecological validity, it introduces external variables that may have influenced accuracy. Additionally, only one Apple Watch measurement was collected per participant, preventing an analysis of intra-subject variability, which would have strengthened reliability conclusions.

**RESPONSE:** We have implemented revisions to the discussion section of the manuscript in response to your comments regarding the composition of the sample, the Apple Watch data collection process, and the fact that one estimate was collected per participant. The limitations section of the discussion has been expanded to describe this with additional detail:

“However, the predominance of participants with high levels of cardiorespiratory fitness represents the study’s most significant limitation. Only one participant was classified as having ‘Fair’ cardiorespiratory fitness, while all others were categorised as ‘Good’, ‘Excellent’, or ‘Superior’. This limits the generalisability of our findings to populations with lower fitness levels, including clinical cohorts. Additionally, the sample primarily consisted of younger individuals, despite efforts to recruit participants across a broad age range. Another limitation is the uncontrolled procedure of generating Apple Watch VO_2_ max estimates. While this protocol reflects real-world use, it introduces unquantified external variables that may have influenced prediction accuracy. Lastly, only one VO_2_ max estimate was collected per participant, preventing analysis of intra-subject variability, which may have provided insights into reliability and changes in measurement accuracy over time.

[...] Future research should include individuals with diverse cardiorespiratory fitness levels to enhance generalisability, support the refinement of predictive algorithms, and develop frameworks for ongoing evaluation of wearable devices. Such efforts are essential to bridging the gap between technological innovation and evidence-based practice, ultimately enhancing the utility of wearable devices for individual health monitoring and public health applications.”

--

Overall, the manuscript is significantly improved and provides valuable insights into the accuracy of Apple Watch VO₂ max estimates. The study is timely and well-conducted, contributing to the growing field of wearable health technology validation. However, to further refine the manuscript, the authors should explicitly emphasize the study’s limited generalizability and acknowledge the need for future research incorporating multiple Apple Watch estimates per participant. With these minor refinements, the manuscript is suitable for publication.

**RESPONSE:** We do hope that we have effectively addressed your comments and suggestions. We endeavoured to emphasise the limited generalisability of our findings to individuals with low cardiorespiratory fitness, whilst highlighting limitations relating to the data collection, the absence of intra-subject variability analysis, and the need for future research to address these limitations. Thank you for your valuable contributions to this manuscript.

--

**Reviewer 2**

**RESPONSE:** The second reviewer indicated in Question 1 that all of their comments from the previous round of review were adequately addressed and that the manuscript is now acceptable for publication.
